# Vitamin B6 and Diabetes: Relationship and Molecular Mechanisms

**DOI:** 10.3390/ijms21103669

**Published:** 2020-05-23

**Authors:** Elisa Mascolo, Fiammetta Vernì

**Affiliations:** Department of Biology and Biotechnology “Charles Darwin”, Sapienza University, 00185 Roma, Italy; elisa.mascolo@uniroma1.it

**Keywords:** vitamin B6, diabetes, AGEs

## Abstract

Vitamin B6 is a cofactor for approximately 150 reactions that regulate the metabolism of glucose, lipids, amino acids, DNA, and neurotransmitters. In addition, it plays the role of antioxidant by counteracting the formation of reactive oxygen species (ROS) and advanced glycation end-products (AGEs). Epidemiological and experimental studies indicated an evident inverse association between vitamin B6 levels and diabetes, as well as a clear protective effect of vitamin B6 on diabetic complications. Interestingly, by exploring the mechanisms that govern the relationship between this vitamin and diabetes, vitamin B6 can be considered both a cause and effect of diabetes. This review aims to report the main evidence concerning the role of vitamin B6 in diabetes and to examine the underlying molecular and cellular mechanisms. In addition, the relationship between vitamin B6, genome integrity, and diabetes is examined. The protective role of this vitamin against diabetes and cancer is discussed.

## 1. Vitamin B6: Roles and Synthesis

Vitamin B6 is a very important compound for general cellular metabolism [1]. The term vitamin B6 refers to six common forms or vitamers, namely pyridoxine (PN), pyridoxal (PL), pyridoxamine (PM), and their related 5′-phosphate derivatives (PNP, PLP, and PMP). The biologically active form, the pyridoxal 5′-phosphate (PLP), acts as coenzyme in about 150 distinct enzymatic activities that catalyze crucial metabolic reactions, such as synthesis, transformation, and degradation of amines and amino acids, supply of one carbon units, transsulfuration, synthesis of tetrapyrrolic compounds (including heme) and polyamines, biosynthesis, and degradation of neurotransmitters [2,3]. Although vitamin B6 is not classified as a classical antioxidant compound, it is able to quench oxygen reactive species (ROS) [4] and counteract the formation of advanced glycation end products (AGEs), genotoxic compounds associated with senescence, and diabetes [5]. Furthermore, PLP works as a modulator of transcription factors, has a role in enzyme folding, and can bind to steroid receptors, playing a role in membrane transport [6].

**Figure 1:**
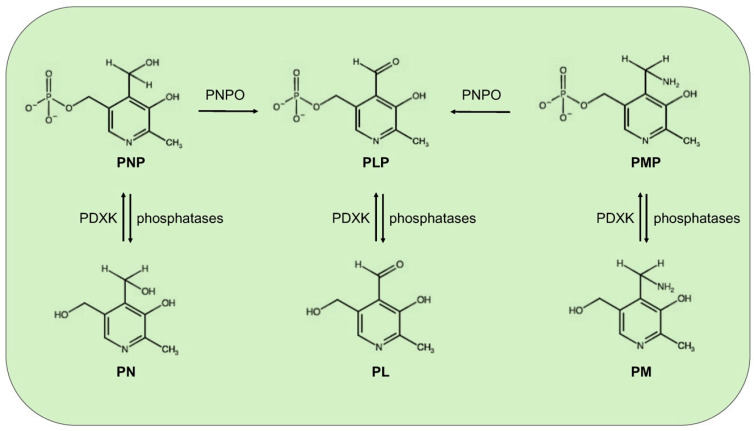
Schematic vitamin B6 metabolism in humans. The diagram corresponds to the pyridoxal 5′-phosphate salvage pathway. PLP, pyridoxal 5′-phosphate; PNP, pyridoxine 5′-phosphate; PMP, pyridoxamine 5′-phosphate; PL, pyridoxal; PN, pyridoxine; PM, pyridoxamine; PDXK, pyridoxal kinase; and PNPO, pyridoxine 5′-phosphate oxidase.

Mammals, different from microorganisms, are not able to synthesize PLP but recycle it through a salvage pathway from B6 vitamers as pyridoxal (PL), pyridoxamine (PM), and pyridoxine (PN) contained in food [7]. In the cytoplasm PL, PM, and PN are converted into the 5′-phosphorylated vitamers through pyridoxal kinase (PDXK), while the FMN-dependent pyridoxine 5′-phosphate oxidase (PNPO) converts PNP and PMP into PLP (Figure 1). Once ingested, PLP, PNP, and PMP are dephosphorylated by the tissue-non-specific alkaline phosphatase (TNSALP), which is anchored to the cell membrane. Then, PM, PN, and PL are absorbed from the upper small intestine by a carrier-mediated system and is delivered to the liver. Here, they are converted to PLP, thanks to the combined action of PDXK and PNPO. From the liver, PLP bound to albumin, along with dephosphorylated B6 vitamers, reach the peripheral tissues through the blood stream. In order to enter cells, PLP needs to be dephosphorylated again by TNSALP [8]. In the cytosol, a ubiquitous PLP phosphatase is instead required for vitamin B6 catabolism [9].

Vitamin B6 recommended dietary allowance is 1.3 mg day^-1^ for adults; it is present in several foods including meat, fish, poultry, vegetables, and fruits, thus, a severe deficiency of this vitamin is uncommon in developed countries, within any diet. However, PLP concentrations appear to be reduced in certain contexts, as for example, alcoholism [10], obesity [11], and pregnancy [12]. Moreover, some pathological conditions such as end-stage renal diseases, chronic renal insufficiency, and other kidney diseases can lower the vitamin B6 levels [12]. In addition, vitamin B6 deficiency has been correlated to malabsorption syndromes, such as celiac disease and inflammatory bowel diseases [12,13,14]. Even the intake of certain medications, including isoniazid [15], penicillamine [16], and cycloserine [17], as well as oral contraceptives, can reduce PLP availability [18]. PLP levels can also be decreased by the inflammation conditions and stress hormones [19,20].

## 2. Vitamin B6 and Diabetes

By considering the plethora of reactions in which vitamin B6 is involved, it is not surprising that its deficiency has been implicated in several clinically relevant diseases, including autism, schizophrenia, Alzheimer, Parkinson, epilepsy, Down’s syndrome, diabetes, and cancer; however, the underlying mechanisms remain unknown in most cases [21,22,23].

Diabetes mellitus (DM) represents a global health problem, touching more than 400 million people and consists of a group of metabolic disorders characterized by persistent hyperglycemia arising from impaired insulin secretion, insulin action, or both [24]. DM is a multifactorial disease caused by the concerted action of genetic and environmental factors, and on the basis of its etiology, it can be classified into three major types—type1 (T1D), type2 (T2D), and gestational diabetes (GDM). T1D is an autoimmune disorder that leads to the destruction of pancreatic beta-cells and accounts for only 5–10% of all diabetes. T2D, the more frequent form (90–95%), is mainly caused by insulin resistance consisting of a diminished tissue response to insulin that leads glucose to accumulate in blood. Consequently, the rate of insulin secretion increases to meet the body’s needs, but this overload, in the long-term, compromises pancreas functionality. GDM is a common pregnancy complication that affects approximately 14% of pregnancies worldwide. It is associated with insulin resistance, in turn generated by a combined action of pregnancy hormones and other factors [25].

Substantial evidence correlates vitamin B6 to diabetes and its complications. Some population screenings have been carried out to compare PLP levels in diabetic groups vs. healthy subjects; in addition, several studies focused on the impact of vitamin B6 on diabetic complications and others on the effectiveness of vitamin B6 as a preventive treatment. Vitamin B6 levels are commonly assessed by measuring plasma pyridoxal 5′-phosphate (PLP) concentration and an inadequate vitamin B6 status is generally associated with a concentration, under the cut-off level of 30 nmol/L. Other methods include the measurement of plasma pyridoxal or total vitamin B6 and urinary 4-pyridoxic acid, as well the ratio between PLP and PL [26]. By examining the studies reported in literature, an inverse relation between vitamin B6 levels and diabetes emerges. Satyanarayana and coworkers [27] in a cross-sectional case-control study found that the mean plasma PLP levels were significantly lower in T2D subjects, compared to the healthy controls. By comparing the results obtained in a Korean study by Ahn and coworkers [28] to those obtained by Nix and collaborators [29] in a German cohort, vitamin B6 levels appeared to be inversely related to the progression of diabetes. Ahn and collaborators [28], in fact, examined diabetic people with an early status of the disease, finding a mean plasma PLP level reduction to be relevant but not statistically significant, with respect to controls; in contrasts, the diabetic group examined by Nix [29], being composed of people with advanced clinical stage, exhibited median plasma concentrations of PLP, PN, and PL that were significantly decreased in a diabetic group compared to the controls. Interestingly, median plasma levels of the PM, PMP, and pyridoxic acid were significantly higher in the diabetes groups than in the controls; this finding led Nix and collaborators to advance the hypothesis that T2D might be associated with an altered activity of the enzymes involved in the interconversion of B6 vitamers [29]. In another study, based on the evidence of increased urinary clearance of vitamin B6, it was hypothesized that decreased vitamin B6 levels in T2D subjects could derive from an impaired reabsorption processes [30]. The same inverse relationship between B6 levels and diabetes was observed in experimental animals [31,32]. Roger was the first to describe decreased PLP levels in streptozotocin-diabetic rats accompanied by less storage in the liver of the mitochondrial PLP [31].

Decreased PLP levels have also be associated with GDM. In a study performed in a group of women affected by GDM, Bennink and Schreurs [33] found that 13 out of 14 displayed reduced PLP levels. Moreover, pyridoxine administration ameliorated oral glucose tolerance. Analogous results were obtained by Spellacy and coworkers [34], which found a clear blood glucose decrease and a normalization of insulin secretion following pyridoxine therapy in GDM women, indicating that vitamin B6 might ameliorate plasma insulin biological activity.

Other intervention studies reported that pyridoxine supplementation is capable of lowering blood glucose levels in streptozotocin-treated rats [35], as well as glycosylated hemoglobin levels in T2D patients [36]. Moreover, Kim and collaborators [37] showed that vitamin B6 can reduce postprandial blood glucose levels following sucrose and starch ingestion, by inhibiting the activity of small-intestinal α-glucosidases.

## 3. Is Reduced Vitamin B6 Availability the Cause or the Effect of Diabetes?

Although an evident link exists between vitamin B6 and diabetes, it is not clear whether the diabetic status is responsible for decreasing PLP availability or, in contrast, whether reduced PLP levels represent a causative agent of diabetes. By examining the literature, it appears that both hypothesis might be plausibly true, suggesting the existence of a vicious circle that correlates vitamin B6 and diabetes. In this paragraph, we reported some evidence in support of each hypothesis by just mentioning some underlying mechanisms that were proposed. In Section 4, we examine in greater details, the mechanisms through which more experimental data converge.

### 3.1. Diabetes Decreases Vitamin B6 Levels

The first evidence that diabetes can reduce PLP levels was provided by Leklem and Hollenbeck [38] who demonstrated that the ingestion of glucose by healthy subjects caused a reduction of PLP levels. In agreement with this idea, Okada and coworkers [32] proposed that diabetes might lead to vitamin B6 deficiency as a result of an increased rate of protein metabolism, due to a diet low in carbohydrates and rich in proteins. Since PLP is cofactor for many enzymes involved in protein metabolism, an increased PLP demand would cause a decrease of PLP in other tissues. This hypothesis is based on the finding that the amount of PLP was found increased in the liver of streptozotocin-diabetic rats, with respect to the nondiabetic controls, but was reduced in the plasma, kidney, and muscles [32]. Accordingly, in diabetic rats fed a low PLP diet, the activity of aspartate amino transferase, an enzyme that is PLP-dependent and is involved in the protein metabolism, was found to be four times greater in the liver of diabetic, as compared to the non-diabetic controls. In contrast, the activity of another PLP-dependent enzyme, the glycogen phosphorylase, was decreased in the muscles of diabetic rats as compared to non-diabetic animals, although the regulation of this enzyme in the muscles also depended on many other factors.

Epidemiological, clinical, and experimental studies have indicated an association between low-grade inflammation and both T1D [39] and T2D [40,41]. Moreover, the role of inflammation in the pathogenesis of T2D and vascular complications was confirmed by intervention studies [41]. In diabetic patients, an inverse relationship between plasma PLP and inflammation markers was found [29]. Therefore, it was proposed that in diabetes, the decline in PLP levels might be due to (1) the mobilization of this coenzyme to the site of inflammation; (2) increased demand by the PLP-dependent enzymes involved into the tryptophan kynurenine pathway; and (3) immune cell proliferation [42]. However, more research is needed to confirm these mechanisms.

### 3.2. Reduced Vitamin B6 Levels Trigger Diabetes

A cause–effect relation between low PLP levels and diabetes emerged from the work of Toyota et al. [43], which showed that pyridoxine deficiency can impair insulin secretion in rats. In addition, by performing in vitro experiments of pancreas perfusion, the authors also found that insulin and glucagon secretion was impaired in the pyridoxine deficiency.

Low vitamin B6 levels are believed to cause GDM, based on the consideration that in pregnancy, PLP levels tend to decline due to the movement of pyridoxine to fetus and, in addition, on the finding that pyridoxine treatment ameliorates glucose tolerance in GDM women [34].

Reduced vitamin B6 availability might also contribute to the appearance of pancreatic islet autoimmunity in T1D. This idea is based on the consideration that PLP is a cofactor for glutamic acid decarboxylase (GAD-65), which represents an important autoantigen implicated in the pathogenesis of T1D. It was hypothesized that reduced levels of the coenzyme might trigger autoimmunity by altering stability, tridimensional conformation, or antigenicity of GAD65 [44].

The most direct evidence indicating that vitamin B6 deficiency could cause diabetes was provided by data from studies in *Drosophila*, showing that mutations in genes involved in vitamin B6 metabolism cause diabetes [45,46].

#### Mutations in Genes Involved in Vitamin B6 Synthesis Cause Diabetes

In pathophysiological studies, cause–effect relationships at the basis of a given disease can be inferred by examining the effects of mutations in the involved genes. This approach is difficult to pursue in human research but is widely used in model organisms. *Drosophila melanogaster*, a successful organism for genetic and cytogenetic studies [47], in the last 10 years, turned out to be a powerful resource to study metabolic diseases, given that the flies share 75% of genes with humans, as well as major metabolic pathways. Both T1D and T2D were modeled in flies and diabetic hallmarks, such as hyperglycemia, altered lipid metabolism, reduced body dimensions, and obesity, were extensively described [48,49,50].

Interestingly, in *Drosophila*, the impact of mutations on diabetes was analyzed in the genes involved in the vitamin B6 salvage pathway, such as *pyridoxal kinase (dPdxk)* and *pyridoxine 5′-phosphate oxidase (sgll).* These studies revealed that mutations in the *dPdxk* gene caused a significant increase in the glucose content of the larval hemolymph (the human blood) [45]. Moreover, the finding that insulin signaling is reduced in the *dPdxk* mutant larvae, suggested that *dPdxk* mutants might represent a new model of T2D [45]. In agreement with these results, the silencing of *sgll* by RNA interference produced diabetic hallmarks, such as hyperglycemia, reduced body size, and altered lipid metabolism [46]. Moreover, vitamin B6 administration rescues diabetic phenotypes in both Pdxk and Sgll depleted individuals, whereas the treatment with the PLP inhibitor 4-deoxypyridoxine (4-DP) causes hyperglycemia in wild type individuals [45,46,51].

Studies aimed at correlating the expression of *PDXK* or *PNPO* human genes with diabetes are still scarce, but encouraging. Moreno-Navarrete and coworkers [52] demonstrated that reduced *PDXK* expression impacts the lipid metabolism (see Section 4.2), raising the possibility that vitamin B6 in obesity can protect from insulin resistance. Moreover, our group also found a link between human *PDXK* gene and diabetes. We demonstrated that the expression, in *dPdxk^1^* mutant flies, of 4 PDXK variants with impaired catalytic activity or affinity for substrates was unable to rescue the hyperglycemia due to *dPdxk^1^* mutation, from the wild-type PDXK protein [53].

## 4. Mechanisms Underlying the Link between Vitamin B6 Diabetes

By considering that PLP is involved in a plethora of metabolic reactions by working as a coenzyme, as well as antioxidant molecule, it is plausible that reduced vitamin B6 levels can impact different diabetic contexts, through different mechanisms. In the Section 3, some hypotheses concerning the mechanisms that relate vitamin B6 to diabetes are mentioned. In this section, the pathways on which most studies converge are analyzed in more detail.

### 4.1. Vitamin B6 and Tryptophan Metabolism

**Figure 2:**
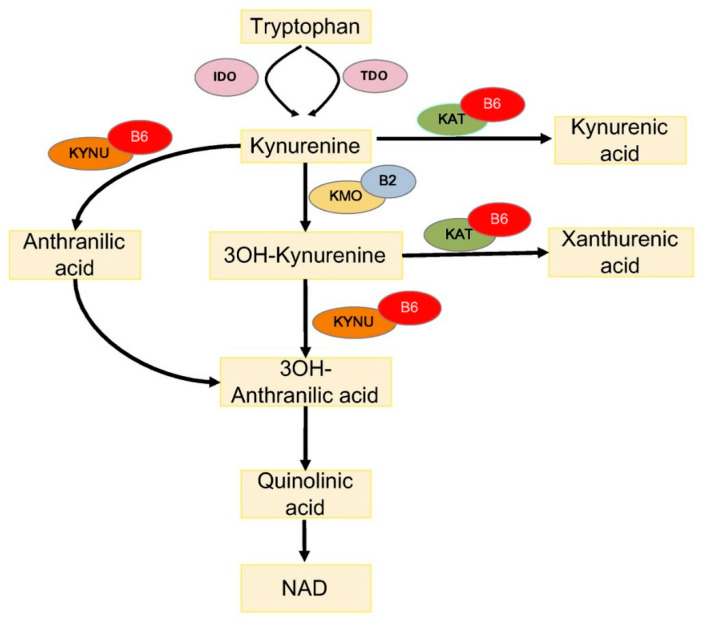
Tryptophan metabolism via the kynurenine pathway. IDO, indoleamine 2,3-dioxygenase; TDO, tryptophan 2,3-dioxygenase; KAT, kynurenine aminotransferase; KMO, kynurenine 3-monooxygenase; KYNU, kynureninase; 3OH-kynurenine, 3-hydroxy kynurenine; 3OH-anthranilic acid, 3-hydroxyanthranilic acid; B6, vitamin B6 (pyridoxal 5′-phosphate); and B2, vitamin B2 (flavin adenine dinucleotide).

One way through which PLP impacts diabetes concerns the metabolism of tryptophan (TRP), an essential amino acid, which is a substrate for the biosynthesis of menthoxyindoles, such as serotonin, N-acetylserotonin, and melatonin. About 95% of TRP is metabolized through the kynurenine (KYN) pathway to produce NAD [54] (Figure 2). TRP- or indoleamine-2,3-dioxygenases (TDO or IDO) convert TRP to KYN and the activity of these enzymes is a rate-limiting step, increased by stress hormones or inflammatory factors (e.g., IFNG and LPS) [55]. KYN is then converted in 3-hydroxykynurenine (3-HKYN), through the action of KYN-monooxygenase (KMO). KYN and 3-HKYN can be converted, respectively, in kynurenic acid (KYNA) and xanthurenic acid (XA), through the activity of the aminotransferases (KAT), which is a PLP-dependent enzyme. The conversion of 3-HKYN into the 3-hydroxyanthranilic acid (3-HAA) is performed by kynureninase (KYNU), which also depends on PLP for its activity. As KYNU is more sensitive to deficiency of PLP, with respect to KAT [56], PLP deficiency diverts 3-HKYN metabolism from the formation of 3-HAA, to accumulation of KYNA and XA [57,58,59,60].

Evidence exists that the tryptophan metabolism is impaired in the different forms of diabetes, due to many factors and conditions, such as pregnancy, oral contraceptives, and emotional and metabolic stress that reduce PLP availability [33,61]. Accordingly, it was found that XA was excessively excreted in diabetic patients [62,63]. Analogously, GDM women after an oral load of TRP exhibited increased XA excretion reduced by PLP administration [33]. Similar results came from animal studies. It was shown that streptozotocin-diabetic rats on TRP treatment excreted much more XA and other TRP metabolites, compared to non-diabetic rats [64] and, more recently, metabolomic studies reported increased KYNA levels in the urine of nonhuman primate and T2D mouse models [65]. Moreover, evidence exists that the KYN pathway is activated in obesity [66] and XA levels have been found to be elevated in pre-diabetic status [67], suggesting that the TRP pathway impairment might contribute to insulin resistance, which precedes T2D [68].

Interestingly, XA has diabetogenic properties because its administration to rats induced diabetic symptoms, worsened by B6 deficiency, including pathological modifications of the pancreatic beta cell tissue [69]. It was proposed that the TRP metabolites impact diabetes through different mechanisms, which can compromise either the insulin activity or insulin secretion. TRP metabolites can be responsible for the (1) formation of chelate complexes with insulin (XA–In), which have about a 50% lower activity compared to pure insulin [70]; (2) formation of Zn++ ion–insulin complexes in β cells that cause a toxic effects on the isolated pancreatic islets [63,71]; (3) inhibition of insulin release from the rat pancreas [72], and (4) induction of pathological apoptosis of pancreatic beta cells [73].

Altered TRP metabolisms is associated with diabetic complications. An elevation in the concentrations of tryptophan metabolites and IDO expression was evident in diabetic retinopathy patients [74]. Moreover, a significant inverse association between toxic TRP metabolites and the stages of chronic kidney diseases, which occur as diabetes complications, was reported [75].

### 4.2. Vitamin B6 and Lipid Metabolism

Reduced vitamin B6 availability can also impact insulin resistance through the lipid metabolism. It was shown that in obese people, adipogenesis and lipotossicity can promote insulin resistance [76,77]. Contrary to previous knowledge that adipogenesis ceases early in the life with a fixed number of adipocytes, the fat cells experience a dynamic process of turnover through which the adipocytes differentiate from pre-adipocyte into mature adipocytes. During adipogenesis, a shift in the gene expression replaces the transcripts proper of the adipocyte early stage with the transcripts responsible for the final maturation [78]. It was shown that adipocyte maturation is compromised in T2D. Larger adipocytes, but similar number of fat cells, were found in diabetic individuals compared to non-diabetic people; moreover, insulin sensitivity was shown to be inversely related to fat cell size. Furthermore, the expression level of some genes involved in adipogenesis was shown to be reduced in T2D subjects, compared to obese non-diabetic individuals [79].

Vitamin B6 is involved in adipogenesis. First evidence come from works in rat models that showed that a vitamin B6-deficient diet significantly reduced adipose tissue and lipogenesis [80,81,82]. Later, it was shown that vitamin B6 administration increased intracellular lipid accumulation in 3T3-L1 adipocytes [83], and reduced macrophage infiltration and adipose tissue inflammation in mice [84,85]. Furthermore, it was shown that vitamin B6 is present at low circulating concentrations in obese people [86]. More recent research by Moreno-Navarrete and coworkers [52] provided more clues on the role played by vitamin B6 in adipogenesis. The authors, by examining adipose tissues from obese subjects, found lower PLP levels in visceral adipose tissues vs. subcutaneous ones; accordingly, they found the *PDXK* expression levels to be significantly reduced and associated with that of the adipogenic genes. In addition, they also demonstrated that *PDXK* mRNA levels, during adipocyte differentiation, were reduced by inflammatory conditions. Moreover, the inhibition of the *PDXK* activity (mediated by 4-DP) reduced adipogenic gene expression, during adipocyte differentiation, whereas PLP administration produced the opposite effect [52].

How exactly PLP regulated adipogenes is not fully elucidated. It was proposed that PLP might activate peroxisome proliferator-activated receptor-γ (PPARγ), one of the master nuclear receptor involved in the expression of adipogenesis genes [83]. Alternatively, PLP might conjugate with RIP140, a nuclear transcription factor, by enhancing its co-repressive activity and its physiological function in adipocyte differentiation [87,88]. Moreover, based on the finding that an altered DNA methylation is associated with adipose tissue dysfunction in T2D patients [89], given that PLP is a coenzyme for serine hydrossymethiltranferase (SHMT), vitamin B6 might contribute to maintain the correct methylation pattern.

Vitamin B6 might also impact the lipid metabolism, through different mechanisms. It was proposed that reduced levels of vitamin B6 might increase levels of homocysteine, as PLP is a cofactor for cystathionine-synthase (CBS) and cystathionine-lyase (CGL), which are involved in the metabolism of this compound [90]. Elevated homocysteine levels are associated with obesity; in addition, they can impair endothelial function and lead to lipid accumulation in liver [91,92,93].

The protective role of vitamin B6 against hepatic lipid accumulation is sustained by the evidence that vitamin B6 administration reduced the accumulation of lipids in livers of high-fat diet-fed *Apoe^−/−^* mice [90] and also that patients affected by nonalcoholic fatty liver disease (NAFLD), a metabolic condition strictly linked to insulin resistance [94], exhibited decreased PLP levels [95]. Although the mechanism that links PLP to hepatic lipid accumulation needs further studies, it has also raised the possibility that vitamin B6 levels could impact NAFLD by impairing polyunsatured fatty acids (PUFA) interconversion. It was shown that vitamin B6 deficiency can contribute to reduce plasma (n-3) and (n-6) PUFA concentrations in healthy subjects [96].

## 5. Vitamin B6 and Diabetes Complications

People with diabetes have an increased risk of developing several serious health problems; persistent high blood glucose levels can damage heart, blood vessels, eyes, kidney, and nerves. A large body of evidence sustains that oxidative stress, combined with a reduced antioxidant defense, plays a crucial role in diabetic complications [97,98,99].

It is thought that one of the main causes of diabetic complication is the formation of AGEs [100]. In diabetes, AGE accumulation is caused by hyperglycemia, through a spontaneous chemical transformation of amine-containing molecules. Reducing sugars bind covalently free amino groups of proteins, lipids, and guanyl nucleotides in DNA (Maillard reaction) to form adducts (Amadori products), including glyoxal, methylglyoxal, and 3-deoxyglucosone (3-DG). These dicarbonyl compounds, apart from being generated in Maillard reactions, also derive from glucose autoxidation, the polyol pathway, and lipid peroxidation reactions. Since they are highly reactive and potent, glycating agents can generate more AGEs by reacting with proteins, in turn [98]. It was shown that AGE accumulation causes inflammation and destroys the normal structure and function of blood vessels, leading to vascular complications [101]. AGEs can also act by bonding to RAGEs (receptor for advanced glycation end-products) in plasma membrane and this interaction triggers different signaling pathways involved in apoptosis, inflammation, angiogenesis, and vasopermeability [102]. Furthermore, AGEs increase the production of reactive oxygen species (ROS), which impact genome integrity [103,104]. The increase in ROS production can, in turn, promote the production of more AGEs, thereby forming a vicious circle [101].

Some studies associated low B6 levels to diabetic complications, such as neuropathy and retinopathy, in T1D and T2D patients [29,105]. While others reported a significant reduction in neuropathy, after vitamin B6 supplementation [106] and reduced progression of diabetic nephropathy [107,108]. Horikawa and coworkers [109] found a high vitamin B6 intake associated with a lower incidence of diabetic retinopathy, in a cohort of T2D Japanese patients. Similar results were obtained in experimental studies. Protective effects of PM in streptozotocin-diabetic rats are described in secondary complications including retinopathy [110] and nephropathy [111]. Accordingly, it was reported that pyridoxine oral administration reduced kidney injury and dysfunction in fat and fructose (HD)-fed mice [112].

It is thought that vitamin B6 impacts diabetic complications mostly by its role as an antioxidant molecule. Muellenbach and coworker [113] demonstrated that the combined treatment of PM and alpha lipoic acid (a powerful antioxidant molecule) resulted in a substantial reduction of the oxidative stress biomarkers in obese Zucker rats. More recently Abdullah and coworkers [114] demonstrated that PM supplementation in alloxan-induced diabetic rats caused a significant decrease in both oxidative stress parameters and ROS production, and consequently reduced DNA damage.

Regarding the mechanism through which vitamin B6 counteracts AGE formation, it was proposed that PLP might trap the 3-DG, one of the AGE pathway metabolites [107,115]. In vitro experiments showed that incubation with PLP markedly decreased concentration of 3-DG in a dose-dependent manner, whereas PL and PM were less reactive. Other studies demonstrated that PM can form stable complexes with metal ions that catalyze the oxidative reactions associated with the advanced stages of protein glycation cascade [116]. Moreover, a density-functional theory (DFT) study indicated that PM might react with reactive carbonyl compounds, generated as byproducts of protein glycation, thereby, counteracting further protein damage [117]. More recently, Ramis and collaborators [118] also emphasized the importance of the antioxidant role of PM in neutralizing AGE formation.

Besides diabetes complications, there is evidence that AGEs can also contribute to diabetes onset. It was shown that glycated insulin display reduced activity [119]. Moreover, it was also proposed that AGEs can directly damage the pancreatic β-cells, based on the finding that aminoguanidine (AG), an AGE inhibitor, was able to protect islet β-cell function. Other studies proposed that AGEs might impair insulin secretion by inhibiting cytochrome-c oxidase and adenosine triphosphate (ATP) production [120].

## 6. Vitamin B6 and DNA Damage in Diabetes

It is widely accepted that oxidative stress is a major risk factor for onset and progression of diabetes. Many of the common risk factors, including obesity and unhealthy eating habits, contribute to produce an oxidative environment that might increase insulin resistance or impair glucose tolerance. Hyperglycemia, in turn, contributes to progression and maintenance of an overall oxidative environment [121]. Consequently, diabetes was associated with reduced levels of antioxidants, such as GSH, vitamin C, and vitamin E [122], and to a low efficiency of DNA repair systems [123,124].

Evidence indicated that both T1D and T2D diabetic patients exhibit oxidative damage and DNA strand breaks [125,126]. It was shown that tissues from diabetic rats and the urine of T1D and T2D patients have increased levels of 8-Oxo-7,8-dihydro-2′-deoxyguanosine (8-oxodG), a sensitive marker of reactive oxygen species (ROS)-induced DNA damage [127,128,129]. In addition, diabetic patients have a higher frequency of sister chromatid exchange than healthy subjects [130,131]. Another study reported high levels of stable chromosomal aberrations in peripheral lymphocytes associated with T2D, and directly correlated with the risk of early diabetes-related death [131]. Moreover, an elevated frequency of micronuclei was found in T2D patients with no microvascular or macrovascular complications [132]. Recently, micronuclei frequency was positively related to glycated hemoglobin (HbA1c) levels, as well as to fasting plasma glucose in T2D [133].

By considering that PLP is both an antioxidant molecule and a cofactor for enzymes involved in DNA metabolism, it is expected that its reduced availability can contribute to increase DNA damage associated with diabetes. Studies in *Drosophila* and human cell cultures helped to gain evidence in support of this hypothesis, demonstrating that PLP deficiency can cause DNA damage throughout the formation of AGEs [45,46]. We demonstrated in fact that PLP deficiency in *Drosophila* and human cultured cells, trigger the formation of chromosome aberrations (CABs). In particular, mutations in the *pyridoxal kinase* gene (*dPdxk^1^*) as well as the RNAi-induced silencing of *pyridoxine 5′-phosphate oxidase* gene *(sgll)*, produced CABs in brain cells [45,46]. Similarly, CABs were produced in wild-type individuals through PLP inhibitors, such as 4-DP, cycloserine, isoniazid, and penicillamine [45]. Interestingly, in all of these cases, CAB frequency was strongly enhanced by treatment with sugars, such as glucose, fructose, or sucrose, whereas in contrast, sugar treatments did not induce CABs in wild-type brains. In addition to providing the first evidence that correlates vitamin B6, DNA damage, and hyperglycemia, these data [45,46] also indicate that low PLP levels and high glucose might synergize in the process of CAB formation. Notably, PLP-depleted cells accumulated significant levels of AGEs, whose formation is enhanced by glucose treatment [45,46]. Remarkably, the treatment with the antioxidant alpha-lipoic acid, rescued not only AGEs but also CABs [45,46], further highlighting the cause–effect relationship between high glucose and CABs in low PLP conditions. These data allowed building a model, according to which decreased PLP levels induce hyperglycemia, which in turn produces AGEs that are responsible for DNA damage through ROS formation. This model could also be applied to humans, based on the following considerations—(1) the human *PDXK* gene inserted in *dPdxk^1^* mutant flies is capable of reducing hyperglycemia, CABs, and AGE accumulation [45]; (2) RNAi-induced silencing of the human *PDXK* gene produces CABs in fibroblasts and HeLa cells, as well as 4-DP treatment in mock cells [45]; (3) alpha-lipoic acid treatment rescued DNA damage induced by PDXK depletion [45]; (4) the expression in the *dPdxk^1^* mutant flies of 4 PDXK human variants with reduced or impaired catalytic activity did not rescue CABs nor AGEs [53]. Taken together, these data suggest not only that low PLP levels could contribute to produce DNA damage in diabetic cells throughout AGE formation, but more importantly, that diabetic patients need to monitor their PLP content to avoid DNA damage, which is a well-known cancer prerequisite.

## 7. Vitamin B6 Diabetes and Cancer

Diabetic patients have an increased risk for developing different types of tumors, including liver, pancreas, colorectal, and lung cancers, but the underlying mechanisms are not fully elucidated [134,135,136]. Hyperinsulinemia and hyperglycemia were hypothesized to have a role in mediating this association, mainly by promoting cell growth [137]. However, it is thought that hyperglycemia might also impact cancer by triggering DNA damage through AGEs and ROS formation.

Based on the above-mentioned findings obtained in *Drosophila*, by considering that DNA damage caused by PLP deficiency is sugar-sensitive, it is reasonable to suppose that in a given diabetic context, the combined action of reduced PLP availability and high endogenous glucose levels could increase the severity of DNA damage. Evidence supporting this hypothesis was provided by studies in *Drosophila*. It was demonstrated in fact that treatment with 4-DP resulted in much more severe DNA damage in diabetic individuals than in wild-type flies [51]. In particular, brains from two different fly models of T2D displayed 60–80% of CABs (vs. 25% in 4-DP treated wild-type individuals) and accumulated many more AGEs. Additionally, treatment with alpha-lipoic acid rescued both AGEs and CABs, confirming that CABs were largely produced by AGEs [51]. Consequently, extrapolated to humans, this finding indicates that low PLP levels might contribute to increased cancer-risk in diabetic patients. It is possible, in fact, to envisage that in an oxidative environment in which antioxidant defenses and DNA repair are weakened, a decline in the capability to counteract AGEs and ROS induced by low PLP levels, might cause an amplification of genotoxic effects, increasing the risk for developing cancer.

## 8. Conclusions

**Figure 3:**
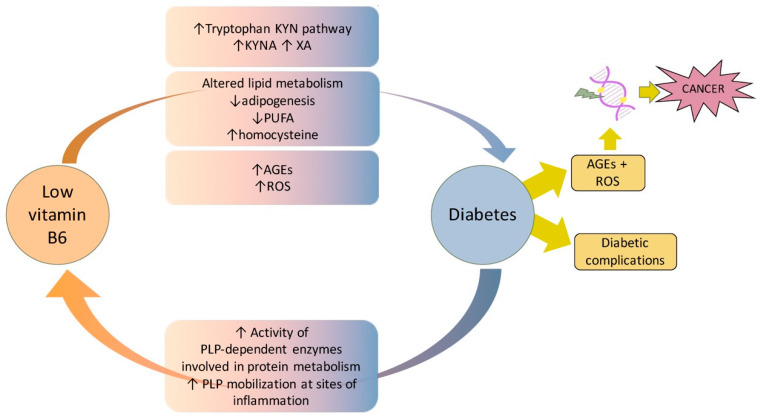
Summary of main mechanisms and pathways at the basis of the association between vitamin B6 and diabetes, inferred from the studies reported in the main text.

In conclusion, compelling evidence demonstrates that vitamin B6 and diabetes are strictly associated through multiple mechanisms and pathways. In T1D, vitamin B6 essentially protects from complications, whereas the hypothesis that it might impact diabetes onset, by triggering autoimmunity, requires further studies. In contrast, the impact of vitamin B6 on T2D is higher due to the different pathophysiology of the two diseases. In T2D, the reduced vitamin B6 availability besides contributing to development of complications can also promote diabetes onset. By examining the literature, it appears that vitamin B6 deficiency can be both a consequence and cause of diabetes (Figure 3). In the first scenario, in contexts including pregnancy and obesity, an increased demand of vitamin B6 from specific PLP-dependent enzymes, as well as the trigger of inflammation pathways, can reduce its availability. In the second scenario, reduced vitamin B6 levels might trigger diabetes onset by impacting the insulin secretion or its biological activity. In this case, mechanisms including enhanced tryptophan catabolism through the kynurenine pathway, reduced rate of adipogenesis, impaired lipid metabolism, or reduced capability to counteract AGE formation, might all contribute to promote diabetes. Furthermore, an impaired antioxidant activity of vitamin B6 can also favor the development of diabetes complications. By also considering the role played by PLP in chromosome integrity maintenance and the high cancer-risk associated with diabetic pathology, it is important to perform further studies to fully clarify molecular mechanisms that link vitamin B6 to diabetes and to confirm in higher organisms the hypothesis that vitamin B6 might contribute to promoting cancer in diabetes. This might allow us to find optimal protocols and strategies to ameliorate diabetes, as well as to decrease cancer-risk in diabetic patients

## Abbreviations

PLP	pyridoxal 5′-phosphate
AGEs	Advanced Glycation End products
CABs	Chromosome aberrations

## References

[1] Hellmann H., Mooney S. (2010). Vitamin B6: A molecule for human health?. Molecules.

[2] Percudani R., Peracchi A. (2003). A genomic overview of pyridoxal-phosphate-dependent enzymes. EMBO Rep..

[3] Di Salvo M.L., Contestabile R., Safo M.K. (2011). Vitamin B(6) salvage enzymes: Mechanism, structure and regulation. Biochim. Biophys. Acta.

[4] Bilski P., Li M.Y., Ehrenshaft M., Daub M.E., Chignell C.F. (2000). Vitamin B6 (pyridoxine) and its derivatives are efficient singlet oxygen quenchers and potential fungal antioxidants. Photochem. Photobiol..

[5] Booth A.A., Khalifah R.G., Todd P., Hudson B.G. (1997). In vitro kinetic studies of formation of antigenic advanced glycation end products (AGEs). Novel inhibition of post-Amadori glycation pathways. J. Biol. Chem..

[6] Lambrecht G., Braun K., Damer M., Ganso M., Hildebrandt C., Ullmann H., Kassack M.U., Nickel P. (2002). Structure-activity relationships of suramin and pyridoxal-5′-phosphate derivatives as P2 receptor antagonists. Curr. Pharm. Des..

[7] McCormick D.B. (1989). Two interconnected B vitamins: Riboflavin and pyridoxine. Physiol. Rev..

[8] Said H.M. (2004). Recent advances in carrier-mediated intestinal absorption of water-soluble vitamins. Annu. Rev. Physiol..

[9] Jang Y.M., Kim D.W., Kang T.C., Won M.H., Baek N.I., Moon B.J., Choi S.Y., Kwon O.S. (2003). Human pyridoxal phosphatase. Molecular cloning, functional expression, and tissue distribution. J. Biol. Chem..

[10] Cravo M.L., Camilo M.E. (2000). Hyperhomocysteinemia in chronic alcoholism: Relations to folic acid and vitamins B(6) and B(12) status. Nutrition.

[11] Ferro Y., Carè I., Mazza E., Provenzano F., Colica C., Torti C., Romeo S., Pujia A., Montalcini T. (2017). Protein and vitamin B6 intake are associated with liver steatosis assessed by transient elastography, especially in obese individuals. Clin. Mol. Hepatol..

[12] Merrill A.H., Henderson J.M. (1987). Diseases associated with defects in vitamin B6 metabolism or utilization. Annu. Rev. Nutr..

[13] Kowlessar O.D., Haeffner L.J., Benson G.D. (1964). Abnormal tryptophan metabolism in patients with adult celiac disease, with evidence for deficiency of vitamin B_6_. J. Clin. Investig..

[14] Chiang E.P., Selhub J., Bagley P.J., Dallal G., Roubenoff R. (2005). Pyridoxine supplementation corrects vitamin B6 deficiency but does not improve inflammation in patients with rheumatoid arthritis. Arthritis Res. Ther..

[15] Biehl J.P., Vilter R.W. (1954). Effect of isoniazid on vitamin B6 metabolism; its possible significance in producing isoniazid neuritis. Proc. Soc. Exp. Biol. Med..

[16] Jaffe I.A., Altman K., Merryman P. (1964). The antipyridoxine effect of penicillamine in man. J. Clin. Investig..

[17] Yamada K., Sawaki S., Hayami S. (1957). Inhibitory effect of cycloserine on some enzymic activities related to vitamin B6. J. Vitaminol..

[18] Lussana F., Zighetti M.L., Bucciarelli P., Cugno M., Cattaneo M. (2003). Blood levels of homocysteine, folate, vitamin B6 and B12 in women using oral contraceptives compared to non-users. Thromb. Res..

[19] Oxenkrug G.F. (2010). Tryptophan kynurenine metabolism as a common mediator of genetic and environmental impacts in major depressive disorder: The serotonin hypothesis revisited 40 years later. Isr. J. Psychiatry Relat. Sci..

[20] Midttun O., Ulvik A., Ringdal Pedersen E., Ebbing M., Bleie O., Schartum-Hansen H., Nilsen R.M., Nygård O., Ueland P.M. (2011). Low plasma vitamin B-6 status affects metabolism through the kynurenine pathway in cardiovascular patients with systemic inflammation. J. Nutr..

[21] Di Salvo M.L., Safo M.K., Contestabile R. (2012). Biomedical aspects of pyridoxal 5′-phosphate availability. Front. Biosci..

[22] Merigliano C., Mascolo E., Burla R., Saggio I., Vernì F. (2018). The Relationship Between Vitamin B6, Diabetes and Cancer. Front. Genet..

[23] Contestabile R., di Salvo M.L., Bunik V., Tramonti A., Vernì F. (2020). The multifaceted role of vitamin B(6) in cancer: *Drosophila* as a model system to investigate DNA damage. Open Biol..

[24] American Diabetes Association (2013). Diagnosis and classification of diabetes mellitus. Diabetes Care.

[25] Plows J.F., Stanley J.L., Baker P.N., Reynolds C.M., Vickers M.H. (2018). The Pathophysiology of Gestational Diabetes Mellitus. Int. J. Mol. Sci..

[26] Leklem J.E. (1990). Vitamin B-6: A status report. J. Nutr..

[27] Satyanarayana A., Balakrishna N., Pitla S., Reddy P.Y., Mudili S., Lopamudra P., Suryanarayana P., Viswanath K., Ayyagari R., Reddy G.B. (2011). Status of B-vitamins and homocysteine in diabetic retinopathy: Association with vitamin-B12 deficiency and hyperhomocysteinemia. PLoS ONE.

[28] Ahn H.J., Min K.W., Cho Y.O. (2011). Assessment of vitamin B(6) status in Korean patients with newly diagnosed type 2 diabetes. Nutr. Res. Pract..

[29] Nix W.A., Zirwes R., Bangert V., Kaiser R.P., Schilling M., Hostalek U., Obeid R. (2015). Vitamin B status in patients with type 2 diabetes mellitus with and without incipient nephropathy. Diabetes Res. Clin. Pract..

[30] Iwakawa H., Nakamura Y., Fukui T., Fukuwatari T., Ugi S., Maegawa H., Doi Y., Shibata K. (2016). Concentrations of Water-Soluble Vitamins in Blood and Urinary Excretion in Patients with Diabetes Mellitus. Nutr. Metab. Insights.

[31] Rogers K.S., Higgins E.S., Kline E.S. (1986). Experimental diabetes causes mitochondrial loss and cytoplasmic enrichment of pyridoxal phosphate and aspartate aminotransferase activity. Biochem. Med. Metab. Biol..

[32] Okada M., Shibuya M., Yamamoto E., Murakami Y. (1999). Effect of diabetes on vitamin B6 requirement in experimental animals. Diabetes Obes. Metab..

[33] Bennink H.J., Schreurs W.H. (1975). Improvement of oral glucose tolerance in gestational diabetes by pyridoxine. Br. Med. J..

[34] Spellacy W.N., Buhi W.C., Birk S.A. (1977). Vitamin B6 treatment of gestational diabetes mellitus: Studies of blood glucose and plasma insulin. Am. J. Obstet. Gynecol..

[35] Nair A.R., Biju M.P., Paulose C.S. (1998). Effect of pyridoxine and insulin administration on brain glutamate dehydrogenase activity and blood glucose control in streptozotocin-induced diabetic rats. Biochim. Biophys. Acta.

[36] Solomon L.R., Cohen K. (1989). Erythrocyte O2 transport and metabolism and effects of vitamin B6 therapy in type II diabetes mellitus. Diabetes.

[37] Kim H.H., Kang Y.R., Lee J.Y., Chang H.B., Lee K.W., Apostolidis E., Kwon Y.I. (2018). The Postprandial Anti-Hyperglycemic Effect of Pyridoxine and Its Derivatives Using In Vitro and In Vivo Animal Models. Nutrients.

[38] Leklem J.E., Hollenbeck C.B. (1990). Acute ingestion of glucose decreases plasma pyridoxal 5′-phosphate and total vitamin B-6 concentration. Am. J. Clin. Nutr..

[39] Clark M., Kroger C.J., Tisch R.M. (2017). Type 1 Diabetes: A Chronic Anti-Self-Inflammatory Response. Front. Immunol..

[40] Esser N., Legrand-Poels S., Piette J., Scheen A.J., Paquot N. (2014). Inflammation as a link between obesity, metabolic syndrome and type 2 diabetes. Diabetes Res. Clin. Pract..

[41] Saltiel A.R., Olefsky J.M. (2017). Inflammatory mechanisms linking obesity and metabolic disease. J. Clin. Investig..

[42] Paul L., Ueland P.M., Selhub J. (2013). Mechanistic perspective on the relationship between pyridoxal 5′-phosphate and inflammation. Nutr. Rev..

[43] Toyota T., Kai Y., Kakizaki M., Ohtsuka H., Shibata Y., Goto Y. (1981). The endocrine pancreas in pyridoxine deficient rats. Tohoku J. Exp. Med..

[44] Rubí B. (2012). Pyridoxal 5′-phosphate (PLP) deficiency might contribute to the onset of type I diabetes. Med. Hypotheses.

[45] Marzio A., Merigliano C., Gatti M., Vernì F. (2014). Sugar and chromosome stability: Clastogenic effects of sugars in vitamin B6-deficient cells. PLoS Genet..

[46] Mascolo E., Amoroso N., Saggio I., Merigliano C., Vernì F. (2020). Pyridoxine/pyridoxamine 5′-phosphate oxidase (Sgll/PNPO) is important for DNA integrity and glucose homeostasis maintenance in *Drosophila*. J. Cell. Physiol..

[47] Cipressa F., Romano S., Centonze S., zur Lage P.I., Vernì F., Dimitri P., Gatti M., Cenci G. (2013). Effete, a *Drosophila* chromatin-associated ubiquitin-conjugating enzyme that affects telomeric and heterochromatic position effect variegation. Genetics.

[48] Musselman L.P., Fink J.L., Narzinski K., Ramachandran P.V., Hathiramani S.S., Cagan R.L., Baranski T.J. (2011). A high-sugar diet produces obesity and insulin resistance in wild-type *Drosophila*. Dis. Models Mech..

[49] Alfa R.W., Kim S.K. (2016). Using *Drosophila* to discover mechanisms underlying type 2 diabetes. Dis. Models Mech..

[50] Graham P., Pick L. (2017). *Drosophila* as a Model for Diabetes and Diseases of Insulin Resistance. Curr. Top. Dev. Biol..

[51] Merigliano C., Mascolo E., La Torre M., Saggio I., Vernì F. (2018). Protective role of vitamin B6 (PLP) against DNA damage in *Drosophila* models of type 2 diabetes. Sci. Rep..

[52] Moreno-Navarrete J.M., Jove M., Ortega F., Xifra G., Ricart W., Obis È., Pamplona R., Portero-Otin M., Fernández-Real J.M. (2016). Metabolomics uncovers the role of adipose tissue PDXK in adipogenesis and systemic insulin sensitivity. Diabetologia.

[53] Mascolo E., Barile A., Mecarelli L.S., Amoroso N., Merigliano C., Massimi A., Saggio I., Hansen T., Tramonti A., Di Salvo M.L. (2019). The expression of four pyridoxal kinase (PDXK) human variants in *Drosophila* impacts on genome integrity. Sci. Rep..

[54] Schwarcz R., Bruno J.P., Muchowski P.J., Wu H.Q. (2012). Kynurenines in the mammalian brain: When physiology meets pathology. Nat. Rev. Neurosci..

[55] Oxenkrug G.F. (2007). Genetic and hormonal regulation of tryptophan kynurenine metabolism: Implications for vascular cognitive impairment, major depressive disorder, and aging. Ann. N. Y. Acad. Sci..

[56] Van de Kamp J.L., Smolen A. (1995). Response of kynurenine pathway enzymes to pregnancy and dietary level of vitamin B-6. Pharmacol. Biochem. Behav..

[57] Bender D.A., Njagi E.N., Danielian P.S. (1990). Tryptophan metabolism in vitamin B6-deficient mice. Br. J. Nutr..

[58] Rios-Avila L., Nijhout H.F., Reed M.C., Sitren H.S., Gregory J.F. (2013). A mathematical model of tryptophan metabolism via the kynurenine pathway provides insights into the effects of vitamin B-6 deficiency, tryptophan loading, and induction of tryptophan 2,3-dioxygenase on tryptophan metabolites. J. Nutr..

[59] Yess N., Price J.M., Brown R.R., Swan P.B., Linkswiler H. (1964). Vitamin B6 depletion in man: urinary excretion of tryptophan metabolites. J. Nutr..

[60] Takeuchi F., Tsubouchi R., Izuta S., Shibata Y. (1989). Kynurenine metabolism and xanthurenic acid formation in vitamin B6-deficient rat after tryptophan injection. J. Nutr. Sci. Vitaminol..

[61] Connick J.H., Stone T.W. (1985). The role of kynurenines in diabetes mellitus. Med. Hypotheses.

[62] Hattori M., Kotake Y., Kotake Y. (1984). Studies on the urinary excretion of xanthurenic acid in diabetics. Acta Vitaminol. Enzymol..

[63] Ikeda S., Kotake Y. (1986). Urinary excretion of xanthurenic acid and zinc in diabetes: (3). Occurrence of xanthurenic acid-Zn2+ complex in urine of diabetic patients and of experimentally-diabetic rats. Ital. J. Biochem..

[64] Akarte N.R., Shastri N.V. (1974). Studies on tryptophan-niacin metabolism in streptozotocin diabetic rats. Diabetes.

[65] Patterson A.D., Bonzo J.A., Li F., Krausz K.W., Eichler G.S., Aslam S., Tigno X., Weinstein J.N., Hansen B.C., Idle J.R. (2011). Metabolomics reveals attenuation of the SLC6A20 kidney transporter in nonhuman primate and mouse models of type 2 diabetes mellitus. J. Biol. Chem..

[66] Favennec M., Hennart B., Caiazzo R., Leloire A., Yengo L., Verbanck M., Arredouani A., Marre M., Pigeyre M., Bessede A. (2015). The kynurenine pathway is activated in human obesity and shifted toward kynurenine monooxygenase activation. Obesity.

[67] Manusadzhian V.G., Kniazev Iu A., Vakhrusheva L.L. (1974). [Mass spectrometric identification of xanthurenic acid in pre-diabetes]. Vopr. Meditsinskoi Khimii.

[68] Oxenkrug G. (2013). Insulin resistance and dysregulation of tryptophan-kynurenine and kynurenine-nicotinamide adenine dinucleotide metabolic pathways. Mol. Neurobiol..

[69] Kotake Y. (1955). Xanthurenic acid, an abnormal metabolite of tryptophan and the diabetic symptoms caused in albino rats by its production. J. Vitaminol..

[70] Kotake Y., Ueda T., Mori T., Igaki S., Hattori M. (1975). Abnormal tryptophan metabolism and experimental diabetes by xanthurenic acid (XA). Acta Vitaminol. Enzymol..

[71] Meyramov G., Korchin V., Kocheryzkina N. (1998). Diabetogenic activity of xanturenic acid determined by its chelating properties?. Transplant. Proc..

[72] Rogers K.S., Evangelista S.J. (1985). 3-Hydroxykynurenine, 3-hydroxyanthranilic acid, and o-aminophenol inhibit leucine-stimulated insulin release from rat pancreatic islets. Proc. Soc. Exp. Biol. Med. Soc. Exp. Biol. Med..

[73] Malina H.Z., Richter C., Mehl M., Hess O.M. (2001). Pathological apoptosis by xanthurenic acid, a tryptophan metabolite: Activation of cell caspases but not cytoskeleton breakdown. BMC Physiol..

[74] Munipally P.K., Agraharm S.G., Valavala V.K., Gundae S., Turlapati N.R. (2011). Evaluation of indoleamine 2,3-dioxygenase expression and kynurenine pathway metabolites levels in serum samples of diabetic retinopathy patients. Arch. Physiol. Biochem..

[75] Debnath S., Velagapudi C., Redus L., Thameem F., Kasinath B., Hura C.E., Lorenzo C., Abboud H.E., O’Connor J.C. (2017). Tryptophan Metabolism in Patients with Chronic Kidney Disease Secondary to Type 2 Diabetes: Relationship to Inflammatory Markers. Int. J. Tryptophan Res..

[76] Cnop M. (2008). Fatty acids and glucolipotoxicity in the pathogenesis of Type 2 diabetes. Biochem. Soc. Trans..

[77] Longo M., Zatterale F., Naderi J., Parrillo L., Formisano P., Raciti G.A., Beguinot F., Miele C. (2019). Adipose Tissue Dysfunction as Determinant of Obesity-Associated Metabolic Complications. Int. J. Mol. Sci..

[78] Choe S.S., Huh J.Y., Hwang I.J., Kim J.I., Kim J.B. (2016). Adipose Tissue Remodeling: Its Role in Energy Metabolism and Metabolic Disorders. Front. Endocrinol..

[79] Dubois S.G., Heilbronn L.K., Smith S.R., Albu J.B., Kelley D.E., Ravussin E. (2006). Decreased expression of adipogenic genes in obese subjects with type 2 diabetes. Obesity.

[80] Huber A.M., Gershoff S.N., Hegsted D.M. (1964). Carbohydrate and fat metabolism and response to insulin in vitamin B6-deficient rats. J. Nutr..

[81] Ribaya J.D., Gershoff S.N. (1977). Effects of vitamin B6 deficiency on liver, kidney, and adipose tissue enzymes associated with carbohydrate and lipid metabolism, and on glucose uptake by rat epididymal adipose tissue. J. Nutr..

[82] Radhakrishnamurty R., Angel J.F., Sabry Z.I. (1968). Response of lipogenesis to repletion in the pyridoxine-deficient rat. J. Nutr..

[83] Yanaka N., Kanda M., Toya K., Suehiro H., Kato N. (2011). Vitamin B6 regulates mRNA expression of peroxisome proliferator-activated receptor-γ target genes. Exp. Ther. Med..

[84] Sanada Y., Kumoto T., Suehiro H., Nishimura F., Kato N., Hata Y., Sorisky A., Yanaka N. (2013). RASSF6 expression in adipocytes is down-regulated by interaction with macrophages. PLoS ONE.

[85] Sanada Y., Kumoto T., Suehiro H., Yamamoto T., Nishimura F., Kato N., Yanaka N. (2014). IκB kinase epsilon expression in adipocytes is upregulated by interaction with macrophages. Biosci. Biotechnol. Biochem..

[86] Aasheim E.T., Hofsø D., Hjelmesaeth J., Birkeland K.I., Bøhmer T. (2008). Vitamin status in morbidly obese patients: A cross-sectional study. Am. J. Clin. Nutr..

[87] Huq M.D., Tsai N.P., Lin Y.P., Higgins L., Wei L.N. (2007). Vitamin B6 conjugation to nuclear corepressor RIP140 and its role in gene regulation. Nat. Chem. Biol..

[88] Bird R.P. (2018). The Emerging Role of Vitamin B6 in Inflammation and Carcinogenesis. Adv. Food Nutr. Res..

[89] Nilsson E., Jansson P.A., Perfilyev A., Volkov P., Pedersen M., Svensson M.K., Poulsen P., Ribel-Madsen R., Pedersen N.L., Almgren P. (2014). Altered DNA methylation and differential expression of genes influencing metabolism and inflammation in adipose tissue from subjects with type 2 diabetes. Diabetes.

[90] Liu Z., Li P., Zhao Z.H., Zhang Y., Ma Z.M., Wang S.X. (2016). Vitamin B6 Prevents Endothelial Dysfunction, Insulin Resistance, and Hepatic Lipid Accumulation in Apoe (−/−) Mice Fed with High-Fat Diet. J. Diabetes Res..

[91] Meigs J.B., Jacques P.F., Selhub J., Singer D.E., Nathan D.M., Rifai N., D’Agostino R.B., Wilson P.W. (2001). Fasting plasma homocysteine levels in the insulin resistance syndrome: The Framingham offspring study. Diabetes Care.

[92] Ala O.A., Akintunde A.A., Ikem R.T., Kolawole B.A., Ala O.O., Adedeji T.A. (2017). Association between insulin resistance and total plasma homocysteine levels in type 2 diabetes mellitus patients in south west Nigeria. Diabetes Metab. Syndr..

[93] Azzini E., Ruggeri S., Polito A. (2020). Homocysteine: Its Possible Emerging Role in At-Risk Population Groups. Int. J. Mol. Sci..

[94] Khoury T., Ben Ya’acov A., Shabat Y., Zolotarovya L., Snir R., Ilan Y. (2015). Altered distribution of regulatory lymphocytes by oral administration of soy-extracts exerts a hepatoprotective effect alleviating immune mediated liver injury, non-alcoholic steatohepatitis and insulin resistance. World J. Gastroenterol..

[95] Li F.J., Zheng S.R., Wang D.M. (2020). Adrenomedullin: An important participant in neurological diseases. Neural Regen. Res..

[96] Zhao M., Lamers Y., Ralat M.A., Coats B.S., Chi Y.Y., Muller K.E., Bain J.R., Shankar M.N., Newgard C.B., Stacpoole P.W. (2012). Marginal vitamin B-6 deficiency decreases plasma (n-3) and (n-6) PUFA concentrations in healthy men and women. J. Nutr..

[97] Brownlee M. (2005). The pathobiology of diabetic complications: A unifying mechanism. Diabetes.

[98] Nowotny K., Jung T., Höhn A., Weber D., Grune T. (2015). Advanced glycation end products and oxidative stress in type 2 diabetes mellitus. Biomolecules.

[99] Forbes J.M., Cooper M.E. (2013). Mechanisms of diabetic complications. Physiol. Rev..

[100] Goldin A., Beckman J.A., Schmidt A.M., Creager M.A. (2006). Advanced glycation end products: Sparking the development of diabetic vascular injury. Circulation.

[101] Yang P., Feng J., Peng Q., Liu X., Fan Z. (2019). Advanced Glycation End Products: Potential Mechanism and Therapeutic Target in Cardiovascular Complications under Diabetes. Oxidative Med. Cell. Longev..

[102] Rowan S., Bejarano E., Taylor A. (2018). Mechanistic targeting of advanced glycation end-products in age-related diseases. Biochim. Biophys. Acta Mol. Basis Dis..

[103] Marques C.M.S., Nunes E.A., Lago L., Pedron C.N., Manieri T.M., Sato R.H., Oliveira V.X.J., Cerchiaro G. (2017). Generation of Advanced Glycation End-Products (AGEs) by glycoxidation mediated by copper and ROS in a human serum albumin (HSA) model peptide: Reaction mechanism and damage in motor neuron cells. Mutat. Res..

[104] Deo P., McCullough C.L., Almond T., Jaunay E.L., Donnellan L., Dhillon V.S., Fenech M. (2020). Dietary sugars and related endogenous advanced glycation end-products increase chromosomal DNA damage in WIL2-NS cells, measured using cytokinesis-block micronucleus cytome assay. Mutagenesis.

[105] Ellis J.M., Folkers K., Minadeo M., VanBuskirk R., Xia L.J., Tamagawa H. (1991). A deficiency of vitamin B6 is a plausible molecular basis of the retinopathy of patients with diabetes mellitus. Biochem. Biophys. Res. Commun..

[106] Cohen K.L., Gorecki G.A., Silverstein S.B., Ebersole J.S., Solomon L.R. (1984). Effect of pyridoxine (vitamin B6) on diabetic patients with peripheral neuropathy. J. Am. Podiatry Assoc..

[107] Nakamura S., Li H., Adijiang A., Pischetsrieder M., Niwa T. (2007). Pyridoxal phosphate prevents progression of diabetic nephropathy. Nephrol. Dial. Transplant..

[108] Elbarbary N.S., Ismail E.A.R., Zaki M.A., Darwish Y.W., Ibrahim M.Z., El-Hamamsy M. (2020). Vitamin B complex supplementation as a homocysteine-lowering therapy for early stage diabetic nephropathy in pediatric patients with type 1 diabetes: A randomized controlled trial. Clin. Nutr..

[109] Horikawa C., Aida R., Kamada C., Fujihara K., Tanaka S., Tanaka S., Araki A., Yoshimura Y., Moriya T., Akanuma Y. (2019). Vitamin B6 intake and incidence of diabetic retinopathy in Japanese patients with type 2 diabetes: Analysis of data from the Japan Diabetes Complications Study (JDCS). Eur. J. Nutr..

[110] Stitt A., Gardiner T.A., Alderson N.L., Canning P., Frizzell N., Duffy N., Boyle C., Januszewski A.S., Chachich M., Baynes J.W. (2002). The AGE inhibitor pyridoxamine inhibits development of retinopathy in experimental diabetes. Diabetes.

[111] Degenhardt T.P., Alderson N.L., Arrington D.D., Beattie R.J., Basgen J.M., Steffes M.W., Thorpe S.R., Baynes J.W. (2002). Pyridoxamine inhibits early renal disease and dyslipidemia in the streptozotocin-diabetic rat. Kidney Int..

[112] Chiazza F., Cento A.S., Collotta D., Nigro D., Rosa G., Baratta F., Bitonto V., Cutrin J.C., Aragno M., Mastrocola R. (2017). Protective Effects of Pyridoxamine Supplementation in the Early Stages of Diet-Induced Kidney Dysfunction. Biomed. Res. Int..

[113] Muellenbach E.A., Diehl C.J., Teachey M.K., Lindborg K.A., Archuleta T.L., Harrell N.B., Andersen G., Somoza V., Hasselwander O., Matuschek M. (2008). Interactions of the advanced glycation end product inhibitor pyridoxamine and the antioxidant alpha-lipoic acid on insulin resistance in the obese Zucker rat. Metabolism.

[114] Abdullah K.M., Abul Qais F., Hasan H., Naseem I. (2019). Anti-diabetic study of vitamin B6 on hyperglycaemia induced protein carbonylation, DNA damage and ROS production in alloxan induced diabetic rats. Toxicol. Res..

[115] Nakamura S., Niwa T. (2005). Pyridoxal phosphate and hepatocyte growth factor prevent dialysate-induced peritoneal damage. J. Am. Soc. Nephrol..

[116] Adrover M., Vilanova B., Frau J., Muñoz F., Donoso J. (2008). The pyridoxamine action on Amadori compounds: A reexamination of its scavenging capacity and chelating effect. Bioorganic Med. Chem..

[117] Ortega-Castro J., Adrover M., Frau J., Salvà A., Donoso J., Muñoz F. (2010). DFT studies on Schiff base formation of vitamin B6 analogues. Reaction between a pyridoxamine-analogue and carbonyl compounds. J. Phys. Chem. A.

[118] Ramis R., Ortega-Castro J., Caballero C., Casasnovas R., Cerrillo A., Vilanova B., Adrover M., Frau J. (2019). How Does Pyridoxamine Inhibit the Formation of Advanced Glycation End Products? The Role of Its Primary Antioxidant Activity. Antioxidants.

[119] Hunter S.J., Boyd A.C., O’Harte F.P., McKillop A.M., Wiggam M.I., Mooney M.H., McCluskey J.T., Lindsay J.R., Ennis C.N., Gamble R. (2003). Demonstration of glycated insulin in human diabetic plasma and decreased biological activity assessed by euglycemic-hyperinsulinemic clamp technique in humans. Diabetes.

[120] Vlassara H., Uribarri J. (2014). Advanced glycation end products (AGE) and diabetes: Cause, effect, or both?. Curr. Diabetes Rep..

[121] Rains J.L., Jain S.K. (2011). Oxidative stress, insulin signaling, and diabetes. Free Radic. Biol. Med..

[122] Bravi M.C., Armiento A., Laurenti O., Cassone-Faldetta M., De Luca O., Moretti A., De Mattia G. (2006). Insulin decreases intracellular oxidative stress in patients with type 2 diabetes mellitus. Metabolism.

[123] Blasiak J., Arabski M., Krupa R., Wozniak K., Zadrozny M., Kasznicki J., Zurawska M., Drzewoski J. (2004). DNA damage and repair in type 2 diabetes mellitus. Mutat. Res..

[124] Zhong A., Chang M., Yu T., Gau R., Riley D.J., Chen Y., Chen P.L. (2018). Aberrant DNA damage response and DNA repair pathway in high glucose conditions. J. Cancer Res. Updates.

[125] Goodarzi M.T., Navidi A.A., Rezaei M., Babahmadi-Rezaei H. (2010). Oxidative damage to DNA and lipids: Correlation with protein glycation in patients with type 1 diabetes. J. Clin. Lab. Anal..

[126] Tatsch E., Bochi G.V., Piva S.J., De Carvalho J.A., Kober H., Torbitz V.D., Duarte T., Signor C., Coelho A.C., Duarte M.M. (2012). Association between DNA strand breakage and oxidative, inflammatory and endothelial biomarkers in type 2 diabetes. Mutat. Res..

[127] Umemura T., Sai K., Takagi A., Hasegawa R., Kurokawa Y. (1990). Formation of 8-hydroxydeoxyguanosine (8-OH-dG) in rat kidney DNA after intraperitoneal administration of ferric nitrilotriacetate (Fe-NTA). Carcinogenesis.

[128] Dandona P., Thusu K., Cook S., Snyder B., Makowski J., Armstrong D., Nicotera T. (1996). Oxidative damage to DNA in diabetes mellitus. Lancet.

[129] Hinokio Y., Suzuki S., Hirai M., Chiba M., Hirai A., Toyota T. (1999). Oxidative DNA damage in diabetes mellitus: Its association with diabetic complications. Diabetologia.

[130] Binici D.N., Karaman A., Coşkun M., Oğlu A.U., Uçar F. (2013). Genomic damage in patients with type-2 diabetes mellitus. Genet. Couns..

[131] Boehm B.O., Möller P., Högel J., Winkelmann B.R., Renner W., Rosinger S., Seelhorst U., Wellnitz B., März W., Melzner J. (2008). Lymphocytes of type 2 diabetic women carry a high load of stable chromosomal aberrations: A novel risk factor for disease-related early death. Diabetes.

[132] Martínez-Pérez L.M., Cerda-Flores R.M., Gallegos-Cabriales E.C., Dávila-Rodríguez M.I., Ibarra-Costilla E., Cortés-Gutiérrez E.I. (2007). Frequency of micronuclei in Mexicans with type 2 diabetes mellitus. Prague Med. Rep..

[133] Grindel A., Brath H., Nersesyan A., Knasmueller S., Wagner K.H. (2017). Association of Genomic Instability with HbA1c levels and Medication in Diabetic Patients. Sci. Rep..

[134] Mocellin S., Briarava M., Pilati P. (2017). Vitamin B6 and Cancer Risk: A Field Synopsis and Meta-Analysis. J. Natl. Cancer Inst..

[135] Zuo H., Ueland P.M., Midttun Ø., Tell G.S., Fanidi A., Zheng W., Shu X., Xiang Y., Wu J., Prentice R. (2019). Vitamin B6 catabolism and lung cancer risk: Results from the Lung Cancer Cohort Consortium (LC3). Ann. Oncol..

[136] Gylling B., Myte R., Schneede J., Hallmans G., Häggström J., Johansson I., Ulvik A., Ueland P.M., Van Guelpen B., Palmqvist R. (2017). Vitamin B-6 and colorectal cancer risk: A prospective population-based study using 3 distinct plasma markers of vitamin B-6 status. Am. J. Clin. Nutr..

[137] Vigneri P., Frasca F., Sciacca L., Pandini G., Vigneri R. (2009). Diabetes and cancer. Endocr. Relat. Cancer.

